# Trends of calcium silicate biomaterials in medical research and applications: A bibliometric analysis from 1990 to 2020

**DOI:** 10.3389/fphar.2022.991377

**Published:** 2022-10-14

**Authors:** Hua Yin, Xiaoli Yang, Lisi Peng, Chuanchao Xia, Deyu Zhang, Fang Cui, Haojie Huang, Zhaoshen Li

**Affiliations:** ^1^ Department of Gastroenterology, General Hospital of Ningxia Medical University, Yinchuan, China; ^2^ Department of Gastroenterology, Changhai Hospital, Second Military Medical University, Shanghai, China; ^3^ Postgraduate Training Base in Shanghai Gongli Hospital, Ningxia Medical University, Shanghai, China

**Keywords:** calcium silicate, biomaterials, bibliometric analysis, regeneration, biomedical

## Abstract

**Background:** Calcium silicate biomaterials (CSB) have witnessed rapid development in the past 30 years. This study aimed to accomplish a comprehensive bibliometric analysis of the published research literature on CSB for biomedical applications and explore the research hotspot and current status.

**Methods:** Articles related to CSB published in the last three decades (1990–2020) were retrieved from Web of Science Core Collection. The R bibliometrix package and VOSviewer were used to construct publication outputs and collaborative networking among authors, their institutes, countries, journals’ matrices and keywords plus.

**Results:** A total of 872 publications fulfilling the search criteria were included. CSB is mainly reported for bone tissues and dental applications. Among researchers, Chang J from Chinese Academy of Sciences and Gandolfi MG from the University of Bologna are the most productive author in these two fields, respectively. China was the leading contributor to the research on CSB in the medical field. A total of 130 keywords appeared more ten or more times were identified. The term “mineral trioxide aggregate” ranked first with 268 occurrences. The co-occurrence analysis identified three major clusters: CSB in dentistry, bone tissue and vitro bioactivity.

**Conclusion:** Calcium silicate biomaterials have a promising scope for various biomedical applications ranging from regeneration of hard tissues (bone and teeth) to skin, tumor, cardiac muscle and other soft tissues. This study may help researchers further understand the frontiers of the field.

## Introduction

A biomaterial is a special functional material that interacts with a biological system such as replacement or regeneration of tissues to restore function (Williams, 2009). In 1970s, Hench et al., introduced a new group of biomaterials, which encouraged the deposition of a layer of hydroxyl carbonated apatite or hydroxyapatite (HA) layer in simulated body fluids mimicking to the mineral phase of bone (Hench and Paschall, 1973). Ca-Si-based bioactive glasses are third generation biomaterial with characteristics of stimulating cells and regeneration of tissues (Hench and Polak, 2002; Hench and Thompson, 2010). However, due to the complex bioactive glasses system, which contains a variety of elements such as calcium, silicon, phosphorus and so on, in order to clearly explore the core reasons for bioactive glass’s biological activity (Gough et al., 2004), calcium silicate containing bioactive materials have been developed (Sanmartin de Almeida et al., 2018).

In 1991, Professor Kokubo reported the formation of a phosphorite layer on the surface of CaO-SiO_2_ glass without P_2_O_5_, whereas CaO-P_2_O_5_ glass without SiO_2_ did not form phosphorite layer (Kokubo, 1991). The dissolution of calcium and silicate ions present in CS leads to the nucleation of HA and bioactivity (De Aza et al., 1999). Silicon ions promote the osteogenic differentiation of osteoblasts (Fei et al., 2012), and stimulate angiogenesis by the regulation of genes expression and interaction between cells (Shie et al., 2011; Wang et al., 2018a). The CS biomaterials have been widely used for various biomedical application due to its suitable properties such as good biocompatibility, antibacterial, anti-inflammatory, cell differentiation and regeneration (Li et al., 2014; Yu et al., 2019). The porous β-CS in rabbit calvarial defects could promote skull regeneration and bone tissue repair (Xu et al., 2008). Composite hydrogels developed on the basis of CS had excellent antibacterial and fibroblast differentiation effects, which had been widely used in the study of infectious wound healing (Xu et al., 2020). CS composites were also utilized to manage infections and bleeding (Hou et al., 2017). Besides, CS served as the foundation for 3D printing, which has excellent photothermal and drug transport properties that were applied to the treatment of osteosarcoma (Truong et al., 2021). In summary, with the development of CS functions, the research interest in medical applications has increased dramatically.

In addition, calcium silicate cements with dicalcium silicate and tricalcium silicate as main components attracts much attention more than 20 years ago. Calcium silicate cements are known to promote hard tissue regeneration by inducing odontogenic differentiation of various cells including dental pulp cells, apical papilla stem, periodontal ligament cells, and bone marrow derived stroma cells through the formation of a complex network by inducing signaling molecules, pathways, receptors and transcriptional control systems (Li et al., 2014; Jung et al., 2015; Zhang et al., 2015; Wang et al., 2018b). Therefore, CS based cements such as mineral trioxide aggregate (MTA) are widely used in pulp capping, pulp cutting, perforation repair and apical barrier (Prati and Gandolfi, 2015). Therefore, it could be challenging for researchers to have a comprehensive and macro view of the subject due to the diversity of CS in the field of medical research. Untimely analysis of potential research frontiers may hinder other innovations and breakthroughs. Bibliometric analysis can well summarize the research hotspots.

Bibliometrics is a multidisciplinary science that applies mathematical and statistical approaches to quantitatively analyze references, authors, journals, countries, institutions, and other indices, and make a comprehensive evaluation of the trends and focus of a particular topic (Lewison and Devey, 1999; Thompson and Walker, 2015). A bibliometric analysis facilitates systematic and intuitive evolution of various research themes assisting researchers to determine the current prospects and trends, thereby signifying ideas and direction for further directions (You et al., 2021; Zhong et al., 2021). Although calcium silicate biomaterials (CSB) have witnessed rapid development in the past 30 years, there is no published bibliometric analysis of the global literature about CSB yet. The aim of the present study was to accomplish a comprehensive bibliometric analysis of the published research literature about CSB over the past three decades.

## Materials and methods

### Data sources and literature search strategy

**FIGURE 1:**
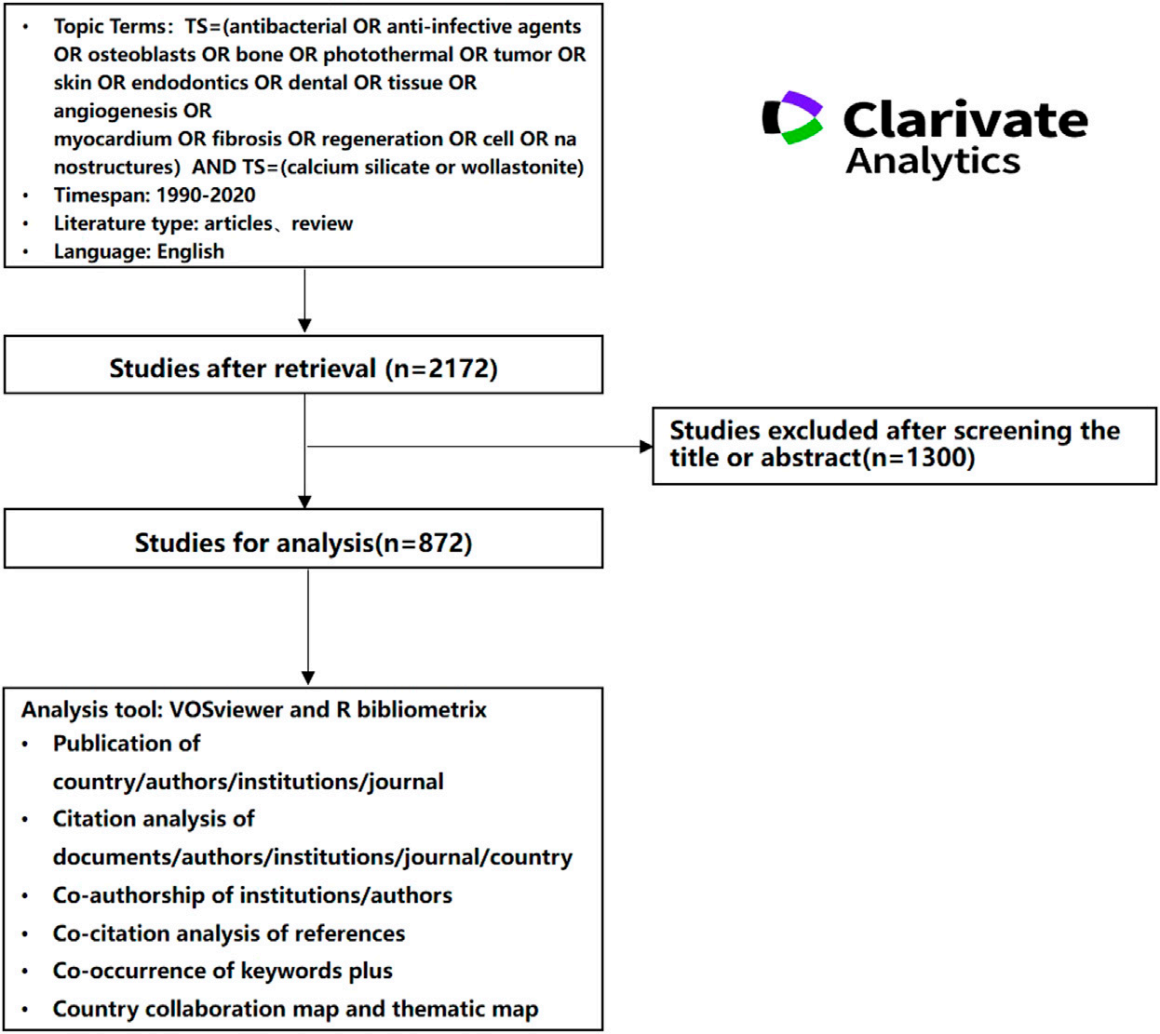
Flow diagram of identification of relevant research.

The Web of Science Core Collection (WoSCC) is the world’s influential multidisciplinary index database of academic literature and serves as a commonly used source of data for conducting a bibliometric study. In the present study, all the data were retrieved from WoSCC on 9 October 2021. The search strategy was as follows: Topic = (antibacterial OR anti-infective agents OR osteoblasts OR bone OR photothermal OR tumor OR skin OR endodontics OR dental OR tissue OR angiogenesis OR myocardium OR fibrosis OR regeneration OR cell OR nanostructures) AND Topic = (calcium silicate OR wollastonite). In order to guarantee the representativeness of the included literatures, the inclusion criteria set as follows: 1) meet the retrieval requirements and the time span was from 1990 to 2019, 2) document type were article and review, 3) publish language was English. Exclusion criteria: 1) non-medical related studies of calcium silicate biomaterials, 2) Document types such as letters, conference abstracts, retracted publications and book chapters were excluded, 3) duplicate publications. Finally, a total of 872 articles were downloaded from the WoSCC, and analyzed (Figure 1).

### Analysis tools

A variety of bibliometric tools have been developed and frequently used, such as VOSviewer, CiteSpace, Bibliometrix R package, BibExcel, etc (Ke et al., 2020). The application of these tools enables beginners and non-bibliometric professional researchers to easily assessed the current research status and hot spots of the topic. Bibliometrix R package was an open-source research bibliometric tool with major bibliometry testing methodologies and was simple to use. Since it was programmed in R, it can be flexibly and quickly upgraded and integrated with other statistical R packages (Aria and Cuccurullo, 2017), but the analysis of cooperative relationships among institutions and authors was insufficient. VOSviewer is a freely available computer program that has been used to construct and view bibliometric maps. VOSviewer focuses more on the graph of bibliometric mapping than commonly used tools like SPSS and Pajek, and it interprets the data in the form of more comprehensible graphics (van Eck and Waltman, 2010). Citespace is characterized by the detection of strong citation references to identify key topics, which is similar to VOSviewer and R packages in bibliometric analysis function, but Citespace operation is relatively complex (Chen, 2006). Bibexcel analysis function is relatively single. Based on the above reasons, we finally choose Bibliometrix R package and VOSviewer for bibliometric analysis. We analyzed the bibliometric data of annual publications, country of publications and total citations, map of cooperation between countries, author impact, journal publications and other factors using the bibliometrix package (R 4.1.0). The citation impact and productivity of researchers’ publications were assessed using the Hirsch Index (H-index) and G-index. The VOSviewer (version 1.6.10) was used to import the data for bibliometric analysis in terms of authors’ institutions, citations and co-occurrence of keywords (van Eck and Waltman, 2010). In the network maps, different clusters were indicated using different colors and collaborations or co-citations were indicated by connecting lines. Circle size represented the numbers of documents, citations, and keyword occurrences, while thickness of connecting lines represented the strength of the links. All the Journal Impact Factors were extracted from the 2020 edition of the Journal Citation Reports (Clarivate).

## Results

### Bibliometric analysis of global publication trends

**FIGURE 2:**
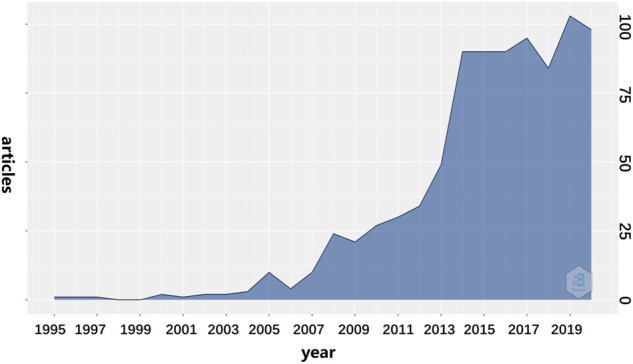
Annual production of CSB in medicine.

A total of 872 publications about the application of CSB in the medicine were retrieved on WoSCC. The evolution of CSB publications over the past 30 years is illustrated in Figure 2. The count of published articles began to grow rapidly after 2012. Although there were fluctuations after that, the overall trend showed a significant increase. In terms of global publication output, there is a static growth in this field with an ingrowth rate of 22.06%/annum conducted by bibliometrix package.

### The publishing performance of countries and institutions

**FIGURE 3:**
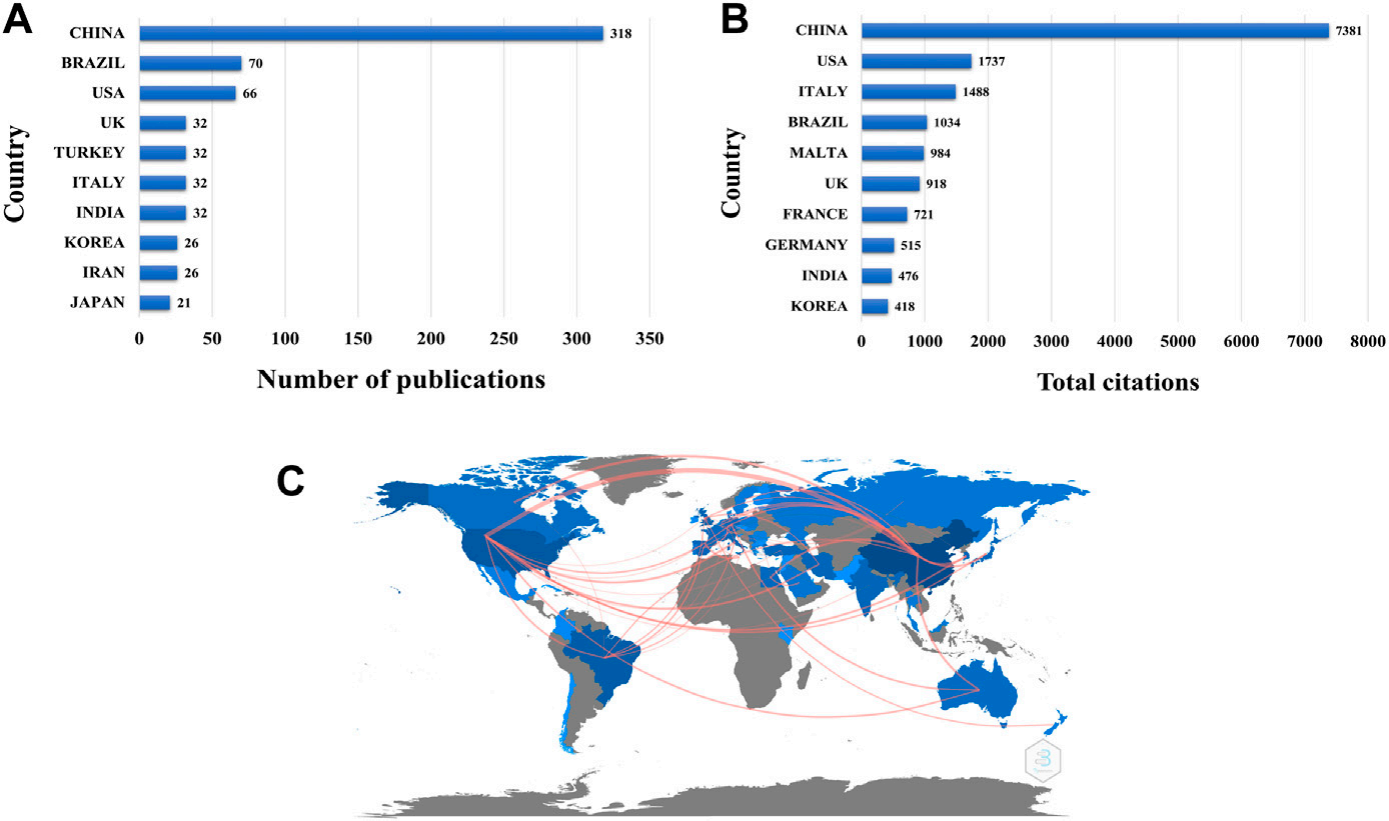
Countries contributing to CSB in medicine field. **(A)** Articles of CSB in medicine field by country/region. **(B)** Total citation country/region ranking of CSB in medicine field. **(C)** World map showing research collaborations among countries/region associated with CSB in medicine. Daker blue color indicates a higher collaboration rate. Countries with more than five shared papers are shown by connectors.

A total of 48 countries and regions contributed to the research of CSB for medical applications. In terms of geographic distribution, China has the largest number of publications in this field, followed by Brazil, United States, United Kingdom (United Kingdom) and Turkey and Figure 3A shows the detailed data. According to the citations data, articles published from China received the highest citations count, followed by United States, Italy, Brazil, and Malta (Figure 3B). The global country/region collaboration map was generated using the Biblioshiny tool (Figure 3C). A total of 146 collaborating countries/regions were found globally, which included the highest number of collaborations (Hirsch, 2005) between China and United States, followed by 9 and 8 collaborations between China and Canada, and between China and Australia (Supplementary Table S1).

**FIGURE 4:**
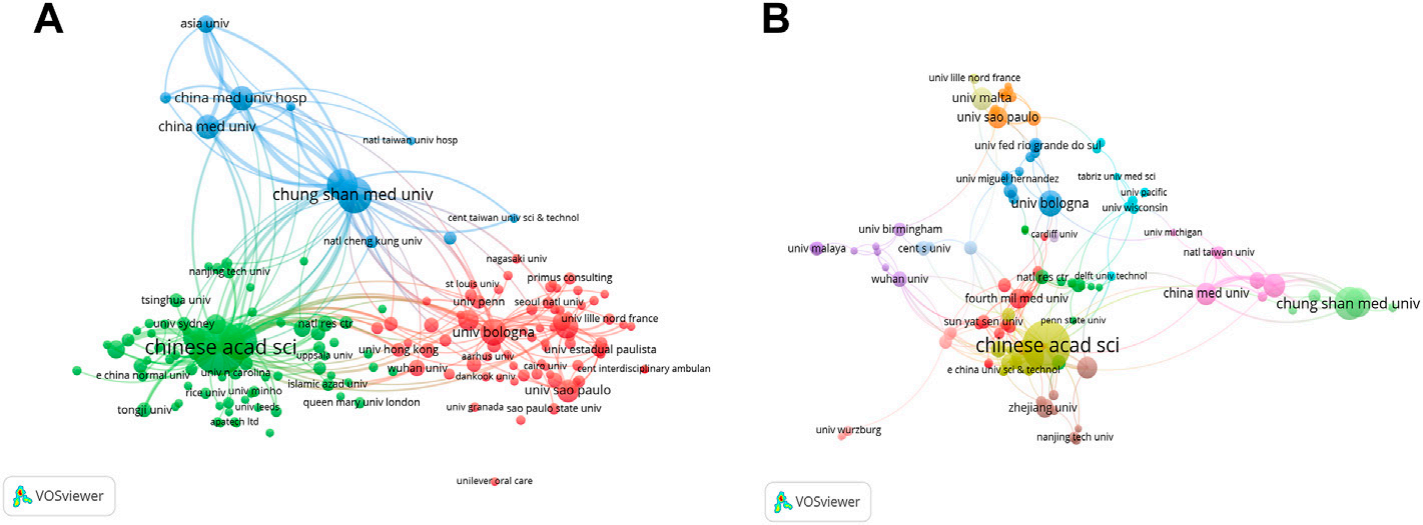
The analysis of institutions. **(A)** Network map of institutions publication about CSB in medicine field by VOSviewer. **(B)** Co-authorship analysis between institutions with more than three publications. The color indicated clusters, circle size indicated number of publications, the thickness of lines indicated strength of linkage.

Researchers from 843 institutions contributed to the field of CSB. Based on the number of publications, 4 institutions lead, namely Shanghai Institute of Ceramics, Chinese Academy of Sciences (113 records, 13.0% of all articles), Shanghai Jiao Tong University (51, 5.8%), Chung Shan Medicine University (47, 5.4%), Chung Shan Medicine University Hospital (39, 4.5%) all of which are located in China. In addition, the University of Bologna (28, 3.2%) in Italy ranks fifth in this field (Figure 4A). The present study analyzed the co-authorship of 156 institutes publishing more than three articles. The exclusion of 33 items that were not connected revealed the collaborations of 123 institutions (Figure 4B). In terms of linking strength, the leading five institutes included Shanghai Institute of Ceramics, Chinese Academy of Sciences (total link strength 109 times), Shanghai Jiao Tong University (71), Chung Shan Medical University (69), Hospital of Chung Shan Medical University (62), and Hospital of China Medical University (51).

### Analysis of journals and authors

The 872 papers were published by 235 different journals. The top ten journals that published the most CSB articles, including the total publications, h-index, most cited journals, total citations and impact factors are shown in Table 1. The Journal of Endodontics published the highest number of articles (80 articles, 9.17%), followed by International Endodontic Journal (50 articles), Materials Science & Engineering C-materials for Biological Applications (38 articles), Dental Materials (34 articles), and Ceramics International (33 articles). Similar trend was observed in terms of citations count, the articles published by the Journal of Endodontics received the highest number of citations, followed by International Endodontic Journal, Dental Materials, Biomaterials, and ACTA Biomaterialia. In terms of journals’ qualitative such as citations, impact factor, and h-index, most of the journals belonged to the first zone of journal citation reports.

**TABLE 1:** Top 10 journal by cited and publication.

Rank	Journal	TC	JCR partition	IF	Journal	H-index	TP
1	Journal of Endodontics	2922	Q1	4.171	Journal of Endodontics	33	80
2	International Endodontic Journal	2020	Q1	5.264	International Endodontic Journal	26	50
3	Dental Materials	1508	Q1	5.304	Materials science & Engineering C-Materials for Biological Applications	18	38
4	Biomaterials	1217	Q1	12.479	Dental Materials	19	34
5	Acta Biomaterialia	801	Q1	8.947	Ceramics International	13	33
6	Materials science & Engineering C-Materials for Biological Applications	778	Q1	7.328	Journal of Materials Science-materials in Medicine	17	26
7	Journal of Materials Science-materials in Medicine	643	Q2	3.896	Journal of Biomedical Materials Research Part B-Applied Biomaterials	13	26
8	Journal of Biomedical Materials Research Part B-Applied Biomaterials	534	Q2	3.368	Journal of Materials Chemistry B	12	20
9	ACS Applied Materials & Interfaces	460	Q1	9.229	ACS Applied Materials & Interfaces	13	16
10	Journal of Materials Chemistry B	440	Q1	6.331	Journal of Biomedical Materials Research Part A	14	16

TC, total citation; JCR, journal citation reports; IF, impact factor; TP, total publication.

A total of 2,897 authors have published papers on CSB for medical applications. The most prolific researcher was Chang J from Chinese Academy of Sciences, with 67 articles (7.7% of all articles), followed by Shie MY (42, 4.81%), Lin KL (27, 3.1%), Gandolfi MG (27, 3.1%), and Prati C (27, 3.1%). In terms of citations count, Chang J received the highest number of citations (2,721), followed by Camilleri J (1,487 citations), Gandolfi MG (1,465 citations), Prati C (1,465 citations), and Shie MY (1,359 citations). The author Chang Jiang ranked first in H index and G index (Table 2). A scientist has index h if h of his or her the number of papers published over n years (Np) have at least h citations each and the other (Np − h) papers have ≤ h citations each. This index can better evaluate the impact of a scientist because it combines the number of publications (number of papers) with the quality (number of citations) (Hirsch, 2005). The G exponent is defined as a set of papers has a g-index g if g is the highest rank such that the top g papers have, together, at least g2 citations. This also means that the top g + 1 papers have less than (g + 1) 2 papers. The G-index is a better way to evaluate those publishing a smaller number of manuscripts with exceedingly high citations, Make up for the h-index (McClelland et al., 2019). This result shows that Author Chang Jiang is one of the most influential scholars in this field.

**TABLE 2:** Publication of the top 10 authors in CSB about medicine field.

Author	H_index	G_index	M_index	TC	TP
Chang J.	28	51	1.647	2721	67
Shie M. Y.	27	35	2.077	1359	42
Gandolfi M. G.	23	27	1.643	1465	27
Prati C.	23	27	1.643	1465	27
Lin K. L.	18	27	1.059	1054	27
Camilleri J.	19	26	1.118	1487	26
Ding S. J.	19	26	1.462	806	26
Huang T. H.	20	24	1.538	689	24
Kao C. T.	18	20	1.385	578	20
Tanomaru M.	12	17	1.5	315	20

TC, total citation; TP, total publication.

**FIGURE 5:**
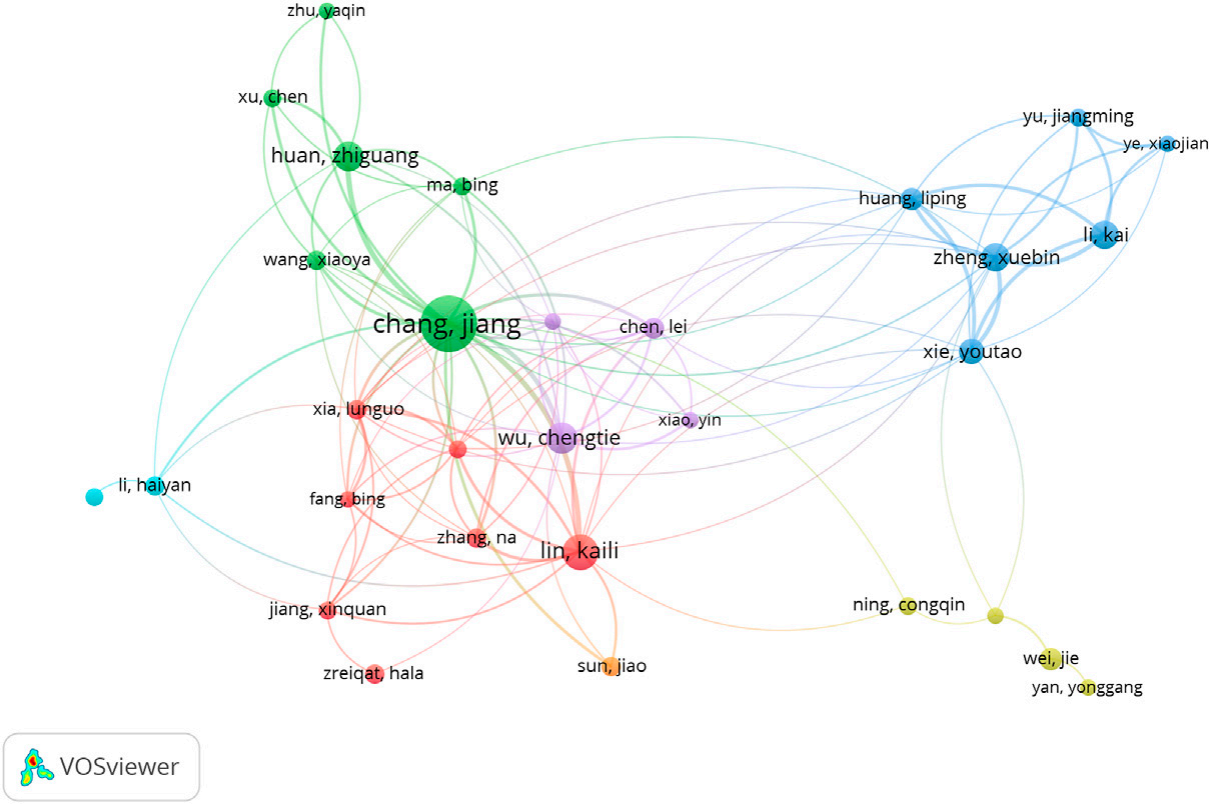
The co-authorship analysis. Network of authorship among authors in CSB in medical research and applications. The color indicated clusters, circle size indicated number of publications, the thickness of lines indicated strength of linkage.

The present study analyzed a total of 86 authors who contributed to eight or more number of publications. The exclusion of 56 items that were not connected revealed the collaborations of 30 authors (Figure 5). The top five most influential authors in terms of total link strength were Chang J (124 times), Shie MY (89 times), Huang TH (65 times), Lin KL (59 times), and Kao CT (56 times).

### Citation and co-citation analyses

**FIGURE 6:**
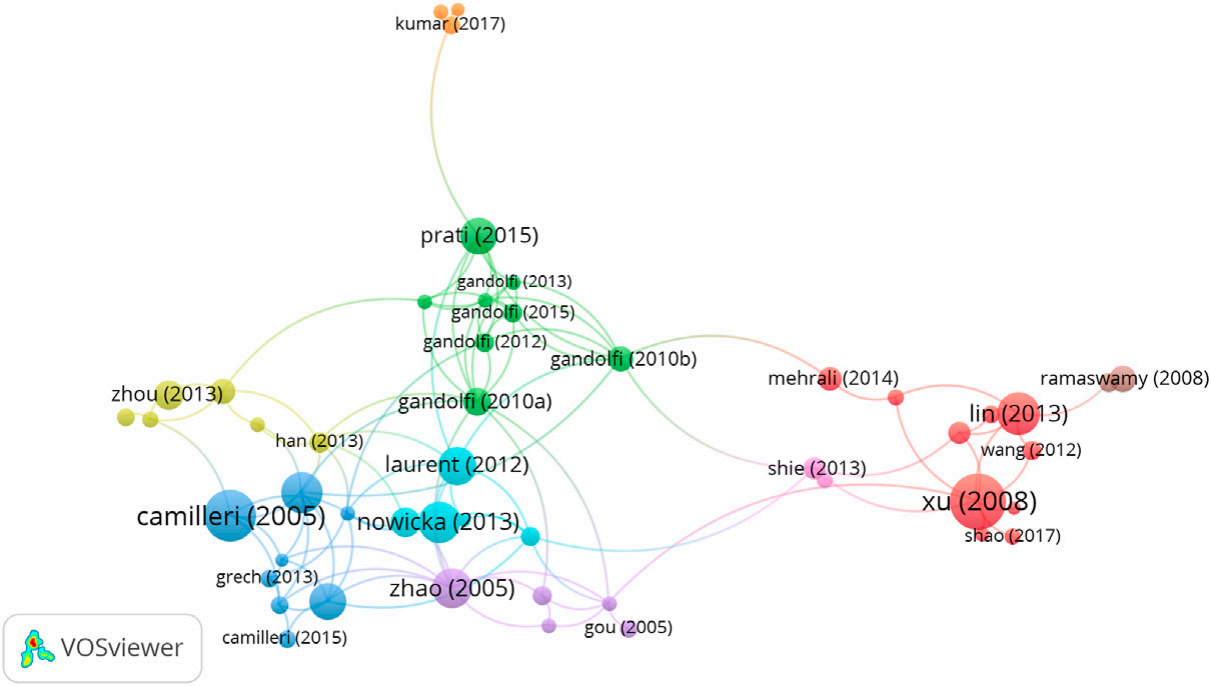
Network map of citation analysis of documents with more than sixty citations.

Based on the citation analysis, 61 received greater than 60 citations (Figure 6). The top ten highly cited articles included “Reconstruction of calvarial defect of rabbits using porous calcium silicate bioactive ceramics” (310 citations) (Xu et al., 2008), followed by “The constitution of mineral trioxide aggregate” (287 citations) (Camilleri et al., 2005), “Enhanced osteoporotic bone regeneration by strontium-substituted calcium silicate bioactive ceramics” (231 citations) (Lin et al., 2013) (Table 3, at the end of the article). Table 3 also lists the top ten references with the highest citations in this field. The review paper by Kokubo T from Chubu University in Japan, published in the Biomaterials, was the most co-cited reference with 109 citations.

**TABLE 3:** TOP 10 article by cited and co-cited reference of CSB in medicine field.

Rank	Author/year/journal	DOI	Total citations	Author/year/journal	DOI	Reference co-citations	Total link strength
1	Xu S., 2008, Biomaterials	10.1016/J.BIOMATERIALS.2008.03.013	310	Kokubo T., 2006, Biomaterials	10.1016/j.biomaterials.2006.01.017	109	577
2	Camilleri J., 2005, Dent Mater	10.1016/J.DENTAL.2004.05.010	287	Parirokh M., 2010, J Endodont	10.1016/j.joen.2009.09.009	85	562
3	Lin K. L., 2013, Biomaterials	10.1016/J.BIOMATERIALS.2013.09.056	231	Xu S., 2008, Biomaterials	10.1016/j.biomaterials.2008.03.013	75	429
4	Camilleri J., 2007, Int Endod J	10.1111/J.1365-2591.2007.01248.X	219	Torabinejad M., 1995, J Endodont	10.1016/s0099-2399(06)80967-2	73	497
5	Nowicka A., 2013, J Endodont	10.1016/J.JOEN.2013.01.005	215	Zhao W. Y., 2005, Biomaterials	10.1016/j.biomaterials.2005.04.025	61	444
6	Zhao W. Y., 2005, Biomaterials	10.1016/J.BIOMATERIALS.2005.04.025	212	Torabinejad M., 2010, J Endodont	10.1016/j.joen.2009.09.010	60	424
7	Laurent P., 2012, Int Endod J	10.1111/J.1365-2591.2011.01995.X	203	Sarkar N. K., 2005, J Endodont	10.1097/01.don.0000133155.04468.41	59	475
8	Prati C., 2015, Dent Mater	10.1016/J.DENTAL.2015.01.004	195	Ni S. Y., 2007, J Biomed Mater Res B	10.1002/jbm.b.30582	50	295
9	Camilleri J., 2013, Dent Mater	10.1016/J.DENTAL.2013.03.007	190	Camilleri J., 2013, Dent Mater	10.1016/j.dental.2013.03.007	49	309
10	Zanini M., 2012, J Endodont	10.1016/J.JOEN.2012.04.018	149	Carlisle E. M., 1970, Science	10.1126/science.167.3916.279	48	317

### Keyword co-occurrence analysis

**FIGURE 7:**
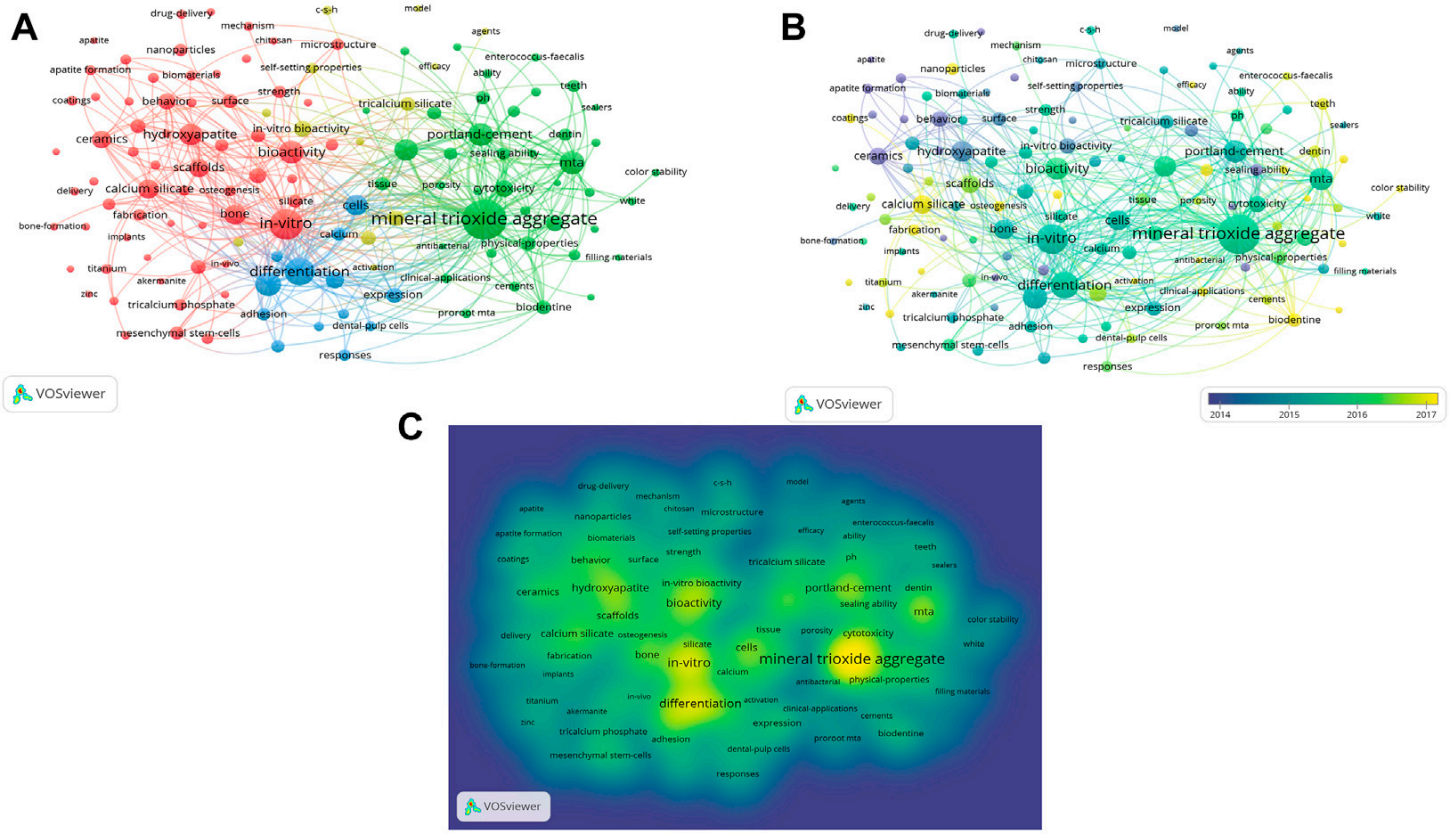
Bibliometric analysis of keywords. **(A)** Map of Clusters based on keywords analysis **(B)** Distribution of keywords plus according to average publication year. The occurrences of theme terms were indicated by the size of the circles. The distance between circles indicated their relationship, different colors indicated variety of clusters **(A)** and average publication year **(B)**. **(C)** Item density visualization of the keywords plus co-citation analysis. Different colors represented the frequency of keyword occurrences, yellow indicated the highest frequency.

The “Keywords Plus” co-occurrence analysis using the VOSviewer revealed 1865 keywords in 872 articles. The present study investigated a total of 130 keywords that were appeared for ten or more times (Figure 7A). The term “mineral trioxide aggregate” appeared most frequently (268 times), followed by “*in vitro*” (157 times), and “differentiation” (131 times). The co-occurrence analysis identified four major clusters: 1) CBS in dentistry, 2) CBS in bone tissue, 3) Effect of calcium silicate biomedical materials on cell function *in vitro*. Keywords published earlier appear in blue and those published late appear in yellow (Figure 7B). Figure 7C is a density visualization map showing the same identification keywords, drawn according to frequency of occurrence.

**FIGURE 8:**
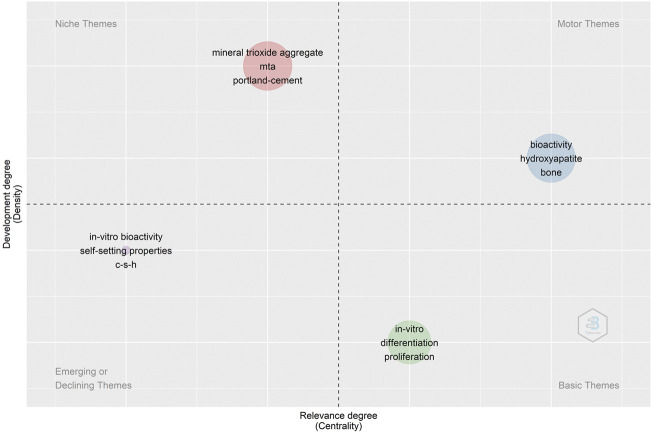
Thematic map of Keywords Plus. The X axis represents the centrality (i.e., the degree of interaction of a network cluster in comparison with other clusters) and gives information about the importance of a theme. The Y axis symbolizes the density (i.e., measures the internal strength of a cluster network, and it can be assumed as a measure of the theme’s development).

Keyword co-occurrence analysis could be considered hot topics within this field. The thematic map formed by two-dimensional matrix can better predict future research directions. The clusters identified by the co-occurrence network are used to draw thematic map according to the centrality and density class values of Callon. In the thematic map, the horizontal and vertical axis represented centrality and density respectively. Accordingly, four quadrants were drawn: the first quadrant (upper right corner): motor-themes, which are well-developed and imperative; the second quadrant (upper left corner): very specialized/niche themes, which are well-developed however not important to the current field; the third Quadrant (lower left corner): fringe themes, which represents some emerging or declining themes/has and a low-density cluster corresponding to “*in vitro* bioactivity,” “self-setting properties’’ and “C-S-H’’. The quadrant 4 (lower right corner) exhibited basic themes, important to the field however not well-developed (Figure 8).

## Discussion

In recent years, CS based bioactive materials have been extensively investigated for a wide range of biomedical application. Although Hou et al. conducted a bibliometric study on biomaterials, the literature search of this study was limited to China and did not include medically related search terms, which could not well reflect the application of biomedical materials in the medical field (Hou et al., 2019). Zhu et al. made a bibliometric analysis of biomaterials in healthcare application, but this study only analyzed the papers published in the journal of advanced healthcare materials in the last 10 years (Zhu et al., 2021). The analysis of biomaterials only from the perspective of this journal may be one-sided. Our present bibliometric study accomplished a comprehensive analysis of the published articles in the field of CSB. In addition, we analyzed the bibliometric data (journals, authors, institutions, countries and distribution of annual output) through network visualizations to understand the current situation. The basis, trends and frontiers of CSB research are discussed using the co-citation and co-occurrence. The overall trend in number of articles published in this field is increasing, especially after 2012 with particularly rapid increases, accounting for 80.2% of the included publications. China remains the leading country in CSB research contributing the highest number of articles, citations, country collaborations, currently leads the world. These findings suggested a vital impact of Chinese institutes and researchers in this field. The Shanghai Institute of Ceramics is the leading Chinese institute that received the top rank in the co-authorship analyses by institution and number of published articles. Remarkably, China established the most cooperation with other countries on the country cooperation map. China has set up dedicated research institutes through new regulations and policies that may have contributed to its leading position. The number of articles published from Brazil, United States, India, and Italy are also increased significantly, accounting for 23.0 percent of all included studies.

Author Change J from Shanghai Institute of Ceramics published the largest number of articles and received the highest citations count, h-index and G-index. These matrices indicated that Change J is an influential scientist in field of CSB. The author Shie MY, from China Medical University Hospital, ranked second. However, the most co-citation reference was published by Japanese author Kokubo T. This suggests that Chinese researchers may need more ground-breaking, basic research to expand the impact. The researchers Gandolfi MG and Prati C (from the University of Bologna, Italy) were ranked third and fourth respectively in terms of publications. They published their research mainly in the endodontic specialty journals indicating their great influence on the applications of CSB in the field of endodontics.

Based on keywords co-occurrence cluster analysis and thematic map, we have summarized the current application status and future development trend of CSB in the medical field. In thematic map, the clusters identified by the co-occurrence network plotted in a thematic map according to Callon’s centrality and Callon’s density rank values along the two axes (Callon et al., 1991). The X-axis represents the centrality, that is, the degree of interaction of a network cluster in comparison with other clusters appearing in the same graph. It can be read as a measure of the importance of a theme in the development of the research field. The Y-axis symbolizes the density, which measures the internal strength of a cluster network, and it can be assumed as a measure of the theme’s development (Cobo et al., 2015). The cluster included keywords, “bioactivity,” “hydroxyapatite,” and “bone” in the first quadrant were characterized by high centrality and high density and were positioned as a motor theme. In the second quadrant, the cluster included keywords, “mineral trioxide aggregate,” “MTA,” and “Portland-cement” were characterized by high density and low centrality as an isolated theme. This represents the application of CSB in dentistry and less sharing important external links with other themes. The cluster was characterized by low centrality and low density and was positioned in the third quadrant, which meant they were weakly developed and marginal. Keywords plus “*in vitro* bioactivity,” “self-setting properties,” and “c-s-h” were related to material properties in the field of dentistry. Considering the development history, we consider it a marginal topic. Another cluster was in the fourth quadrant, characterized by high centrality and a lower density with respect to the two previous clusters. It was a basic and transversal well-developed theme. The most frequent words were “*in vitro*,” “differentiation” and “proliferation”. *In vitro* studies of CSB’s effects on different cells have expanded its range of applications.

### Cluster (1) calcium silicate biomaterials in dentistry: Sealing performance and biocompatibility

CSB represented by MTA is widely used in dentistry because of its excellent biocompatibility, bioactivity and ability of setting in the moistened environment (presence of body fluids such as saliva, blood, dentinal fluid). In addition, CSB had demonstrated a good hermetic sealing ability and radiopacity (Prati and Gandolfi, 2015; Utneja et al., 2015). HA precipitates are generated on the surface of CSB, which penetrate into the dentin tubules to establish chemical bonding with dentin, thus possessing good sealing performance of root canals. Nabeel et al. (2019) confirmed that CSB had a good sealing performance and demonstrated an efficient and lasting sealing ability in the root apical region. Similarly, ProRoot MTA increases clinical applicability with the addition of bismuth oxide to improve radiopacity. However, Gandolfi et al. have shown *in vitro* experiments that bismuth oxide may reduce/impair the biocompatibility of the bone cement (Gandolfi et al., 2009a; Gandolfi et al., 2010). Therefore, CSB free from bismuth oxide had increased biocompatibility (Camilleri and Gandolfi, 2010). MTA is the first CSB used in the dental field, and later new materials were developed to enhance the properties and overcome the shortcomings of the original cements. For example, the setting time of Biodentine is only 9 min, that is significantly fast setting compared to the original cements, MTA Plus with gel (55 min), MTA Angelus (80 min) (Gandolfi et al., 2009b; Gandolfi et al., 2011a; Gandolfi et al., 2014; Gandolfi et al., 2015).

Many studies have demonstrated that traditional and resin-based endodontic sealers have cytotoxicity (Geurtsen and Leyhausen, 1997; Bouillaguet et al., 2006). Therefore, CSB root canal sealers are preferred due to better physicochemical and biological properties (Gandolfi and Prati, 2010). Furthermore, Bryan et al. (2010) investigated the *in vitro* biocompatibility of various endodontic sealers using an osteogenic cell line and demonstrated better compatibility of the experimental CSB compared to the AH sealer. In addition, several *in vitro* studies confirmed that CSB is less cytotoxic when used as pulp capping agents. Brignardello-Petersen et al. summarized the results of 27 studies and found that pulpitis was less likely to occur after MTA direct pulp capping, and restorative dentin bridge was easier to form (Brignardello-Petersen, 2019). Paschos showed that CSB has obvious clinical advantages in indirect pulp capping treatment of deep caries (Paschos, 2014). Additional restorative applications of CSB included pulp therapy in primary teeth (Agamy et al., 2004), dentin hypersensitivity (Gandolfi et al., 2008), root perforation repairing (De-Deus et al., 2012), dentin remineralization (Gandolfi et al., 2011b; Niu et al., 2014), apexification and pulp revascularization (Silujjai and Linsuwanont, 2017; Fang et al., 2018). CSBs are continuously explored for various dental applications and likely to play an increasingly significant role in the field of dentistry in future.

### Cluster (2) calcium silicate biomaterials in bone tissue: Regeneration and mechanical properties

Relevant studies have shown that CS can be transformed into HA in simulated body fluids. After bioactive ceramics are implanted into the human body, bone tissues adhere to, grow and form chemical bonds on the surface of HA (de Aza et al., 1996). Considering CSB has good degradation performance, it participates in the metabolism of human body, and ultimately achieves tissue replacement while using for the bone tissue therapy. However, poor tensile strength and brittleness of CSB limit its applications for load bearing areas in bone repair (Wieding et al., 2014). Further studies have enhanced the performance of CSB by improving the preparation process, doping ions, and compounding with other biomaterials to overcome its shortcomings and expand the application scope (Ge et al., 2019; Mabrouk et al., 2019). The preparation of CSBs by ion doping effectively control the dissolution rate and further improved its osteogenic and mechanical properties (Lu et al., 2010; Zreiqat et al., 2010). Lin et al. (2004) prepared porous CSB with different pore sizes and porosity to improve the mechanical properties. The flexural strength and fracture toughness of CSB may be further reduced through mesoporous structures. 3D-printed silicate/calcium sulfate composite scaffolds demonstrated excellent mechanical strength, biological activity, and drug release ability at targeted sites, which has the potential for the bone regeneration applications (Chen et al., 2019; Zhang et al., 2021). In short, CSB had been widely used in bone tissue by improving their properties.

### Cluster (3) effect of calcium silicate biomedical materials on cell function *in vitro*: Mechanism and pathways

For bone tissue and pulp therapy applications, the interactions between CSB and cells affected cells’ behaviors including their proliferation, differentiation, migration, and apoptosis. The biocompatibility and bioactivity of CSB play significant functions in bone tissue and pulp therapy. *In vitro* evaluation of biocompatibility and bioactivity are very important to elucidate the mechanism of CSB affecting cell behavior, and further explore the functions and applications of CSB.

The MTA *via* the ERK signaling pathway promotes the proliferation and survival of various stem cells in human including DPSCs, PDLSCs, and bone marrow stromal/stem cells (BMSCs) (Chen et al., 2016). In addition, CSBs induce odonto/osteogenic differentiation of stem cells *via* signal molecules, pathways, receptors and transcriptional control systems (Luo et al., 2014; Jung et al., 2015; Tian et al., 2015; Yin et al., 2018). MTA enhances odontogenic/osteogenic differentiation of SCAP by up-regulating inflammatory cytokines (IL-1α, IL-1β, and TNF-α), stimulating I kappa B kinase (IKK) phosphorylation, and translocation of NF-κB subunit (Yan et al., 2014). La-Doped mesoporous CS/chitosan scaffold can induce osteogenic differentiation of BMSC through TGF-β/Smad pathway, and promote the formation of new bone *in vivo* (Peng et al., 2019). Similarly, CSB can also affect cell-cell interactions. Li et al. (2014) indicated that CS significantly stimulated angiogenesis and osteogenic differentiation of co-cultured HUVEC and BMSC through paracrine. Moreover, host immune defense of implants has an important impact on osteogenesis and new bone formation. Huang et al. (2018) reported that CSBs reduced the cell viability and proliferation of macrophages, decreased the secretion of inflammatory cytokines and promoted the caspase-dependent apoptosis of macrophages.

In addition, CBS is also used for research into tumor and other diseases. Nano-structured CSBs are drug delivery materials because of their unique properties including nanoporous/hollow structure, high bioactivity, biodegradability, pH responsive drug release behavior, enhanced loading capacity, and release time thereby significantly prolonging the therapeutic efficacy (Wu et al., 2010; Wu et al., 2013a; Zhu and Sham, 2014). Wu J investigated hybrid nanoparticles of amorphous hydrated CS/block copolymer monomethoxy(polyethyleneglycol)-block-poly(lactide-co-glycolide) (CSHP) and demonstrated an enhanced loading capacity of the anticancer drug docetaxel. In addition, the release of loaded docetaxel was significantly enhanced in phosphate buffered saline (pH 5.5) compared to release rate at pH 7.4, indicating its promising potential for cancer treatment (Wu et al., 2013b). CS based biomaterials that can be further functionalized by specific structural designs or doped with functional components can be used to address wound healing with different needs. Multifunctional composite hydrogel comprised of copper-doped calcium silicate ceramic and polydopamine enhanced the photothermal properties, improved the antibacterial activity against methicillin-resistant *Staphylococcus aureus*, and promoted healing of infectious skin wound (Xu et al., 2020). These modified materials can also enhance the hemostatic effect (Hou et al., 2017). Inspired by the bioactive of CSBs, Yi et al. (2019) demonstrated that CS ionic solution significantly increases cardiomyocytic activity, promotes cell-to-cell communication and improves cardiac function post-myocardial infarction. Wang et al. first reported that Si ion can enhance the adipogenic differentiation of hBMSCs by stimulating the expression of peroxisome proliferator-activated receptor *γ* and other adipogenic differentiation switches, suggesting that Si ion releasing biomaterials have a promising potential for the repair of adipose tissues (Wang et al., 2018a). Another study also found that CS ions reduced pulmonary fibrosis (Chen et al., 2021). In conclusion, the guidance for surface functionalization or modification of CS carriers may enhance the potential applications of CSBs in various biomedical fields. CSBs promote proliferation and differentiation of various cells including fibroblasts, osteoblasts, stem cells, regulate immune cells and soft tissue cells such as myocardium. More mechanisms of CSB action were discovered through *in vitro* experiments, which promoted CSB to be applied in medical fields.

## Future prospect

It can be seen that CSB had gradually begun to be used in anti-tumor, heart, lung and other visceral tissue repair, and good effects had been found, indicating that they may be applied to organ tissue repair in the future. But it was limited in animal research level, and a lot of research is still needed before clinical application. The application of CBS in bone tissue has a long ceramic degradation cycle (Feng et al., 2021), and new biomaterials need to be further developed for the problem of ceramic degradation. In the future, the development of CBS with higher antibacterial properties, promoting the remineralization of diseased dentin and tissue regeneration is the focus. At the same time, more accurate randomized controlled trials or large-scale clinical trials are needed to confirm the clinical practicability of these materials.

### Limitation

There are some limitations of the present study. First, our data are only searched from WoS database, and the results of other databases are not collected. However, it is recognized that WoS database is used for bibliometric analysis. Second, the macro level trends of a research topic should can been available through bibliometric analysis. However, bibliometrics analysis is based on mathematical and statistical techniques. Due to the complex and dynamic nature of databases, it is not possible to completely attain adequate and effective information at the macro level. Third, there is selection bias. Although English is the most frequently used language for academic writing, only considered articles and review publications in English, which may not be fully representative of all the research on CSB.

## Conclusion

We summarized the research status and trend of CSBs for various biomedical applications. CSB can be used in various forms such as powder, nanoparticles, composite materials, self-setting cement, coating. Although the application of silicate biomaterials received more attention for soft tissue regeneration and tumor therapy, their application scope is increasingly wide ranging from the regeneration of hard tissue (bone, tooth) to skin, tumor, cardiac muscle and other soft tissues. The scope of silicate biomaterials is very promising however require further research to improve the properties and performance for various biomedical applications.

## Data Availability

The original contributions presented in the study are included in the article/Supplementary Material, further inquiries can be directed to the corresponding authors.

## Abbreviations

TC, total citation; JCR, Journal Citation Reports; IF, impact factor; TP, total publication.
