# Supplementary material for: Trends of calcium silicate biomaterials in medical research and applications: A bibliometric analysis from 1990 to 2020
Source: Front Pharmacol. 2022 Oct 14;13:991377. doi: 10.3389/fphar.2022.991377 (PMC9614043; doi:10.3389/fphar.2022.991377)
Supplement: Supplementary file 1 [file Table1.DOCX]

**Table S1 Top 10 collaboration countries/regions**

| **From** | **To** | **Frequency** |
| --- | --- | --- |
| CHINA | USA | 30 |
| CHINA | CANADA | 9 |
| CHINA | AUSTRALIA | 8 |
| CHINA | EGYPT | 8 |
| CHINA | KOREA | 8 |
| USA | ITALY | 7 |
| CHINA | JAPAN | 6 |
| FRANCE | MALTA | 6 |
| USA | IRAN | 6 |
| USA | KOREA | 6 |
